# First-in-human phase I clinical trial of the NY-ESO-1 protein cancer vaccine with NOD2 and TLR9 stimulants in patients with NY-ESO-1-expressing refractory solid tumors

**DOI:** 10.1007/s00262-020-02483-1

**Published:** 2020-01-24

**Authors:** Mikiya Ishihara, Yasutaka Tono, Yoshihiro Miyahara, Daisuke Muraoka, Naozumi Harada, Shinichi Kageyama, Takeshi Sasaki, Yasuhide Hori, Norihito Soga, Katsunori Uchida, Taizo Shiraishi, Eiichi Sato, Hideki Kanda, Toshiro Mizuno, Gill A. Webster, Hiroaki Ikeda, Naoyuki Katayama, Yoshiki Sugimura, Hiroshi Shiku

**Affiliations:** 1 Department of Medical Oncology, Mie University Hospital, 2-174 Edobashi, Tsu, Mie 514-8507 Japan; 2 Department of Personalized Cancer Immunotherapy, Mie University Graduate School of Medicine, 1577 Kurimamachiya-cho, Tsu, Mie 514-8507 Japan; 3 Department of Oncology, Nagasaki University Graduate School of Biomedical Sciences, 1-12-4 Sakamoto, Nagasaki, Nagasaki 852-8523 Japan; 4 United Immunity, Co., Ltd., Room220, Mie University Campus Incubator, 1577 Kurimamachiya-cho, Tsu, Mie 514-8507 Japan; 5 Department of Immuno-Gene Therapy, Mie University Graduate School of Medicine, 2-174 Edobashi, Tsu, Mie 514-8507 Japan; 6 Department of Nephro-Urologic Surgery and Andrology, Mie University Graduate School of Medicine, 2-174 Edobashi, Tsu, Mie 514-8507 Japan; 7 Kameyama Nephro-Urologic Clinic, 1488-215 Sakaemachi, Kameyama, Mie 519-0111 Japan; 8 Department of Urology, Aichi Cancer Center Hospital, 1-1 Kanokoden, Chikusa-ku, Nagoya, Aichi 464-8681 Japan; 9 Department of Pathology, Mie University Graduate School of Medicine, 2-174 Edobashi, Tsu, Mie 514-8507 Japan; 10 Department of Pathology, Institute of Medical Science (Medical Research Center), Tokyo Medical University, 6-7-1 Nishishinjuku, Shinjuku-ku, Tokyo, 160-0023 Japan; 11 Innate Immunotherapeutics, Melbourne, VIC 3051 Australia; 12 Department of Hematology and Oncology, Mie University Graduate School of Medicine, 2-174 Edobashi, Tsu, Mie 514-8507 Japan

**Keywords:** Cancer vaccine, NY-ESO-1 antigen, NOD2, TLR9

## Abstract

Cholesteryl pullulan (CHP) is a novel antigen delivery system. CHP and New York esophageal squamous cell carcinoma 1 (NY-ESO-1) antigen complexes (CHP-NY-ESO-1) present multiple epitope peptides to the MHC class I and II pathways. Adjuvants are essential for cancer vaccines. MIS416 is a non-toxic microparticle that activates immunity via the nucleotide-binding oligomerization domain 2 (NOD2) and TLR9 pathways. However, no reports have explored MIS416 as a cancer vaccine adjuvant. We conducted a first-in-human clinical trial of CHP-NY-ESO-1 with MIS416 in patients with NY-ESO-1-expressing refractory solid tumors. CHP-NY-ESO-1/MIS416 (μg/μg) was administered at 100/200, 200/200, 200/400 or 200/600 (cohorts 1, 2, 3 and 4, respectively) every 2 weeks for a total of 6 doses (treatment phase) followed by one vaccination every 4 weeks until disease progression or unacceptable toxicity (maintenance phase). The primary endpoints were safety and tolerability, and the secondary endpoint was the immune response. In total, 26 patients were enrolled. Seven patients (38%) continued vaccination in the maintenance phase. Grade 3 drug-related adverse events (AEs) were observed in six patients (23%): anorexia and hypertension were observed in one and five patients, respectively. No grade 4–5 drug-related AEs were observed. Eight patients (31%) had stable disease (SD). Neither augmentation of the NY-ESO-1-specific IFN-γ-secreting CD8^+^ T cell response nor an increase in the level of anti-NY-ESO-1 IgG1 was observed as the dose of MIS416 was increased. In a preclinical study, adding anti-PD-1 monoclonal antibody to CHP-NY-ESO-1 and MIS416 induced significant tumor suppression. This combination therapy is a promising next step.

**Electronic supplementary material:**

The online version of this article (10.1007/s00262-020-02483-1) contains supplementary material, which is available to authorized users.

## Introduction

Peptide cancer vaccines have been evaluated in previous studies, but they have thus far exhibited limited efficacy against advanced cancers. Adjuvant selection is an important factor that affects the success of cancer vaccines. For example, substances that are retained at the injection site should be avoided as cancer vaccine adjuvants. Hailemichael et al. [1] reported that incomplete Freund’s adjuvant-based vaccination caused T cells to accumulate at the vaccination site rather than at the tumor site and induced T cell apoptosis. Stimulants of pathogen recognition receptors, including TLRs and nucleotide-binding oligomerization domain (NOD)-like receptors, could activate antigen-presenting cells and may thereby overcome the immunosuppressive tumor microenvironment. TLR stimulants, such as OK-432, CpG or poly-ICLC, have been evaluated in conjunction with the New York esophageal squamous cell carcinoma 1 (NY-ESO-1) antigen-related cancer vaccine in clinical studies [2-8]. One of the most promising agents is a stimulant of TLR9. Muraoka et al. [9] reported that immunization with a peptide vaccine without an adjuvant increased apoptosis in vaccine-induced CD8^+^ T cells; in contrast, immunization with a peptide vaccine with a TLR9 stimulant reduced apoptosis in vaccine-induced CD8^+^ T cells and induced a significant anti-tumor effect in a mouse model. The stimulation of multiple innate immunity signaling pathways may greatly improve the efficacy of cancer vaccines over that achieved by TLR9 signaling alone.

MIS416 is a nontoxic microparticle adjuvant derived from *Propionibacterium* acnes that activates the immune response via the NOD2 and TLR9 pathways. MIS416 acts as a Th1 response-skewing adjuvant by promoting the CD8^+^ T cell response and enhancing the anti-tumor activity of vaccines in a mouse model [10]. However, no clinical trials of cancer vaccines with MIS416 as an adjuvant have been reported. The NY-ESO-1 antigen, a cancer-testis antigen, was identified in esophageal cancer by serological expression cloning (SEREX) performed using serum obtained from patients with autologous esophageal squamous cell carcinoma [11, 12]. The NY-ESO-1 antigen is expressed in a variety of cancers; for example, the NY-ESO-1 antigen is expressed in approximately 40% of refractory urothelial cancers [13-15], approximately 15–40% of advanced prostate cancers [16, 17] and 49–75% of synovial cell sarcomas [18, 19] but is not expressed in normal tissues with the exception of the testis and placenta. These findings suggest that the NY-ESO-1 antigen could be an ideal target for cancer immunotherapy against many malignant tumors and may have high cancer specificity and low toxicity. Cholesteryl pullulan (CHP) is a polysaccharide-based novel antigen delivery system for cancer vaccines. A complex of CHP and the NY-ESO-1 antigen (CHP-NY-ESO-1) was constructed that contains multiple MHC class I- and II-restricted epitopes and efficiently induces antigen-specific CD4^+^ and CD8^+^ T cell immunity [20-25]. We conducted single-center, open-label, dose-escalation studies of CHP-NY-ESO-1 with MIS416 as an adjuvant in patients with NY-ESO-1-expressing refractory urothelial cancer or castration-resistant prostate cancer and malignant solid tumors to evaluate its safety, tolerability, and immune response [26, 27].

## Materials and methods

### Mice, cell lines and anti-mouse PD-1 monoclonal antibodies

Female BALB/c mice aged 6–10 weeks were used. A mouse colon tumor cell line CT26 was transfected with human NY-ESO-1 [9, 28]. Tumor volume was calculated as follows: 0.5 × length (mm) × width (mm)^2^. An anti-mouse PD-1 (clone RMP1-14) monoclonal antibody (mAb) was produced in-house from hybridoma and purified as monoclonal [29].

### Patients and treatment

Patients meeting the following criteria were included: histologically documented urothelial cancer, prostate cancer (clinical trial Registration Number UMIN000005246) or malignant solid tumors (UMIN000008006) that were refractory to standard therapy, age ≥ 20 years, an Eastern Cooperative Oncology Group (ECOG) performance status (PS) scale of 0–2, a life expectancy ≥ 3 months, adequate organ function and positive tumor expression of NY-ESO-1. Patients with a history of active autoimmune disease, the use of steroids (more than 20 mg equivalent of prednisolone/day), the use of immunosuppressive drugs, uncontrolled infections or previous NY-ESO-1-related immunotherapy were excluded.

Patients were enrolled from March 2011 to February 2017 and received CHP-NY-ESO-1 (0.5 mg/mL)/MIS416 (2 mg/mL) administered at 100 μg/200 μg, 200 μg/200 μg, 200 μg/400 μg or 200 μg/600 μg (cohorts 1, 2, 3 and 4, respectively) every 2 weeks for a total of 6 doses during the treatment phase (clinical trial registration number UMIN000005246 and UMIN000008006) followed by vaccination every 4 weeks (maintenance phase) until disease progression, patient refusal or unacceptable toxicity (UMIN000008007). CHP-NY-ESO-1 and MIS416 were manufactured according to good manufacturing practices and provided by ImmunoFrontier, Inc. (Tokyo, Japan) and Innate Therapeutics Ltd., respectively. CHP-NY-ESO-1 was subcutaneously (s.c.) injected into the chest, abdomen, upper arm or lower leg. MIS416 was injected s.c. at a site 2 cm away from the periphery of the CHP-NY-ESO-1 injection bulge. Mixing the 2 drugs under the skin was prohibited. The primary endpoints were safety and tolerability, and the secondary endpoints were immune response and quality of life (QOL). The dose-limiting toxicity (DLT) was defined as grade 3 or higher for injection site reaction, allergic reaction, pruritus, chills, and fever. The maximum tolerated dose (MTD) was the highest dose that caused DLT in no more than one of 6 patients. Patients assessable for dose escalation were those who were treated with more than 4 courses. If the number of assessable patients was lower than three, patients were added to the cohort. In cohort 4, 2 of the 4 patients had a total of 3 severe adverse events (SAEs), including 1 treatment-related case of anorexia, 1 nervous system disorder caused by cancer progression and 1 case of pancreatitis caused by alcohol intake. The data and safety committee recommended stopping further patient enrollment in cohort 4 because of the high frequency of SAEs. We decided to terminate the clinical trial considering the long amount of time required for patient enrollment and because no further improvements in efficacy were expected.

### Assessment

Treatment response was assessed at 12 weeks by computed tomography. Patients with prostate cancer were also assessed by bone scans and the detection of serum PSA levels. Responses were assessed according to the Response Evaluation Criteria in Solid Tumors (RECIST) criteria version 1.1 [30]. AEs were assessed according to the National Cancer Institute Common Terminology Criteria for Adverse Events version 4.0. Serial serum and PBMC samples were collected before and during treatment.

QOL was assessed using the European Organization for Research and Treatment of Cancer (EORTC) QLQ-C30 Japanese version 3.0 every 4 weeks as follows: at baseline and on treatment phase course 3 day 1, treatment phase course 5 day 1, and treatment phase course 6 day 15 (clinical trial registration number UMIN000005246). The EORTC QLQ-C30 is a questionnaire that was developed to assess the QOL of cancer patients. The QLQ-C30 contains 5 functional scales (physical, role, cognitive, emotional, and social), 3 symptom scales (fatigue, pain, and nausea and vomiting), and a global health and QOL scale [31].

### Expression of the NY-ESO-1 antigen

Archival or newly obtained tumor samples obtained from patients were screened for NY-ESO-1 expression. Eligible patients were those with NY-ESO-1 expression in ≥ 1% of tumor cells according to immunohistochemical staining with an E978 monoclonal antibody or ≥ 1 copy NY-ESO-1/10^4^ copies of glyceraldehyde-3-phosphate dehydrogenase (GAPDH) according to a quantitative real-time PCR (qRT-PCR) analysis.

### T cell response, NY-ESO-1-specific antibody response and cytokine kinetics

Serum samples were obtained at baseline, 6 h and 1 week after the 1st vaccination, and 2 weeks after each vaccination. PBMCs were obtained at baseline, before the 4th vaccination and 2 weeks after the 6th vaccination. All samples were stored at − 80 °C until analyzed.

The T cell response was assessed by ELISPOT assay as previously described [32]. In brief, ELISPOT plates (MAHA S4510; Millipore) were coated with an anti-human interferon (IFN)-γ monoclonal antibody (R&D Systems, Minneapolis, MN). A total of 5 × 10^4^ sensitized CD4^+^ or CD8^+^ T cells and 1 × 10^5^ peptide-pulsed irradiated CD4^−^ CD8^−^ PBMCs were placed in each well of the ELISPOT plate. NY-ESO-1-overlapping peptides were grouped as follows: anterior half-mix p1–20, p11–30, p21–40, p31–50, p41–60, p51–70, p61–80, p71–90 and p81–100 and posterior half-mix p91–110, p101–120, p111–130, p119–141, p131–150, p139–160, p151–170 and p161–180. After the mixtures were incubated for 22 h at 37 °C, the plate was washed, the biotinylated capture antibody was added, and the combined mixture was incubated overnight at 4 °C. After the cells were washed, they were reacted with streptavidin–alkaline phosphatase conjugate and then stained using an alkaline phosphatase conjugate substrate kit (BioRad, Hercules, CA). The spots were counted using an ELISPOT Plate Reader (ImmunoSpot, CTL-Europe GmbH, Bonn, Germany).

The NY-ESO-1-specific antibody response was assessed by ELISA as previously described [24, 25]. Briefly, recombinant NY-ESO-1 proteins (His-tag and GST-tag) were absorbed onto immunoplates (442,404; Nunc, Roskilde, Denmark) at a concentration of 10 ng/50 μL/well at 4 °C. The collected serum samples were diluted from 1:400 to 1:6400. After washing and blocking the plate, the sera were added and incubated for 10 h. After washing, goat anti-human IgG (H + L chain) (MBL, Nagoya, Japan) conjugated with peroxidase was added. After adding the TMB substrate (Pierce, Rockford, IL), the plate was read using a Microplate Reader (model 550; BioRad, Hercules, CA). Serum samples were obtained from 83 healthy volunteers and assayed by ELISA for the NY-ESO-1 IgG antibody as described in another study performed at Mie University (Mie University approval number: 817) before the start of this clinical study using CHP-NY-ESO-1 and MIS416. The cutoff level selected for the anti-NY-ESO-1 IgG antibody was defined as the mean optical density (OD) 450–550 absorption value + 1.645 × the standard deviation as 0.254. Hence, an OD 450 absorption value of at least 0.254 was considered a positive reaction at a serum dilution of 1:400. For patients who were antibody-positive at the baseline, their antibody titers were judged to be “augmented” if they changed by fourfold or more compared with the baseline. The IgG subclass antibody response to the NY-ESO-1 protein was detected with ELISA using polyclonal sheep anti‑human IgG1 (dilution, 1:25,600; cat. no. AP006), IgG2 (dilution, 12,800; cat. no. AP007) and IgG3 (dilution, 1:12,800; Cat. No. AP008) (H + L chain) conjugated with HRP (The Binding Site Group Ltd., Birmingham, UK) used as the secondary antibodies.

The serum levels of 48 cytokines and chemokines were determined using Bio-Plex kits (Bio-Rad, Hercules, CA) according to the manufacturer's instructions. The kits’ analytes were IL-1α, IL-1β, IL-1Ra, IL-2, IL-2Rα, IL-3, IL-4, IL-5, IL-6, IL-7, IL-8, IL-9, IL-10, IL-12 (p40), IL-12 (p70), IL-13, IL-15, IL-16, IL-17, IL-18, eotaxin, basic fibroblast growth factor (bFGF), G-CSF, GM-CSF, IFN-γ, C–C motif chemokine ligand 2 (CCL2), CCL3, CCL4, CCL5, platelet-derived growth factor (PDGF)-BB, cutaneous T-cell-attracting chemokine (CTACK), growth-related oncogene (GRO)-α, hepatocyte growth factor (HGF), IFNα2, leukemia inhibitory factor (LIF), CCL7, macrophage colony-stimulating factor (M-CSF), macrophage migration inhibitory factor (MIF), C–X–C motif chemokine ligand 9 (CXCL9), CXCL10, β-nerve growth factor (NGF), stem cell factor (SCF), stem cell growth factor (SCGF)-β, stromal cell-derived factor (SDF)-1α, TNF-α, TNF-β, TRAIL and VEGF [33].

### Statistical analysis

The combined data analyses of the two treatment phase trials are described in the protocol. The Mann–Whitney *U* test was used to compare data obtained in the two groups. Fisher's exact test was used to compare the IgG positivity rates of patients who received CHP-NY-ESO-1 at 100 µg versus those who received CHP-NY-ESO-1 at 200 µg. Kruskal–Wallis ANOVA was used to compare data obtained in more than three groups. Student's *t* test was used to assess changes in QLQ-C30 scores between the pretreatment and treatment phases. *P*-values below 0.05 were considered statistically significant. Calculations were performed with SPSS Statistics version 25 (IBM Japan, Ltd., Tokyo, Japan).

## Results

### Patient characteristics and treatment exposure

In total, 26 patients were enrolled (13 with prostate cancer, 5 with urothelial cancer, 4 with synovial sarcoma and 4 with other cancers, as shown in Table 1 and Supplementary Table 1). The median age was 70 years old (range 36–84). All patients had received prior therapies (chemotherapy, radiation, and/or surgery). Eight patients, all of whom were all prostate cancer patients, received systemic dexamethasone (DEX) (Supplementary Table 2). Nine patients were enrolled in cohort 1, 7 in cohort 2, 6 in cohort 3 and 4 in cohort 4. The median number of vaccinations was 6 (range 1–66). Seven patients (38%) moved to the maintenance phase and received ≥ 7 doses of the vaccine.

**Table 1:** Patient characteristics

No. of patients	26
Sex	
Male/female	20/6
Age, median (range)	70 (36–84)
Tumor types	
Prostate	13
Urothelial	5
Synovial sarcoma	4
Other^a^	4
Prior anti-cancer therapy	
Surgery	15
Radiotherapy	7
Chemotherapy	25
Hormonal therapy	13
Systemic steroid use	
Yes/no^b^	8/18

^*^Esophagus, ovary, sarcoma, rectum

^§^1 mg/day: 3 patients, 0.5 mg/day: 4 patients, < 0.5 mg/day: 1 patient

### Drug-related adverse events

The drug-related AEs reported in this study are shown in Table 2. Grade 1–2 injection site reactions were observed in all patients. Grade 3 drug-related AEs were observed in 6 patients (23%): 5 exhibiting hypertension and 1 presented with anorexia. There was 1 case of grade 3 hypertension (11.1%) in cohort 1, 2 (28.6%) in cohort 2, 1 (16.7%) in cohort 3, and 1 (25.0%) in cohort 4 (Supplementary Table 3). The patients with these AEs were all prostate cancer patients and had grade 2 hypertension at baseline. One patient required an increase in the dose of antihypertensive medication, and the other AEs were resolved without medical modification. No grade 4–5 drug-related AEs were observed.

**Table 2:** Drug-related adverse events

*N* = 26	Any grade	Grade 3
Injection site reaction	26	0
Hypertension	8	5
Malaise	3	0
Sinus tachycardia	3	0
LDH increased	3	0
Hyperkalemia	3	0
Hypertriglyceridemia	3	0
Chills	2	0
QTc interval prolongation	2	0
Platelet count decreased	2	0
ALT increased	2	0
Fibrinogen increased	2	0
FDP increased	2	0
Anorexia	1	1

Grade 1 anemia, increased ALP, decreased APTT, increased CPK, dysgeusia, hypokalemia, hyponatremia, infection (herpes zoster virus), decreased lymphocyte count and sore throat were observed in 1 patient each. No grade 4–5 drug-related AEs were observed

*ALT* alanine aminotransferase, *FDP* fibrin degradation products, *LDH* lactate dehydrogenase

### Responses

Eight patients (31%) had stable disease (SD) (Table 3). Among the 13 patients with prostate cancer, 5 had SD (38%), 7 had PD (54%) and 1 was N/A (7%) (Supplementary Table 2). Among these patients, 4 advanced to the maintenance phase. One patient with prostate cancer who was enrolled in cohort 2 received 60 vaccinations during the maintenance phase. He showed no disease progression or significant increase in the serum PSA levels over 5 years.

**Table 3:**
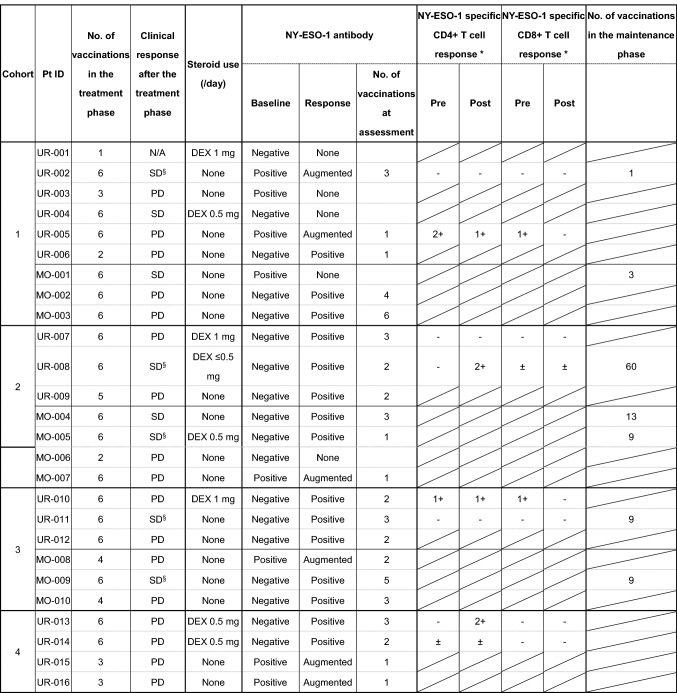
Clinical and immune responses

The number of spots (target - control) in 5 × 10^4^ cells assessed by an ELISPOT assay was graded as follows: –: < 5, ±: 5–10, 1+: 11–50, and 2+: >50

^§^Patients who had no measurable lesion at baseline

*DEX* dexamethasone, *N/A* not assessed

### Serial titers of anti-NY-ESO-1 IgG

**Fig. 1:**
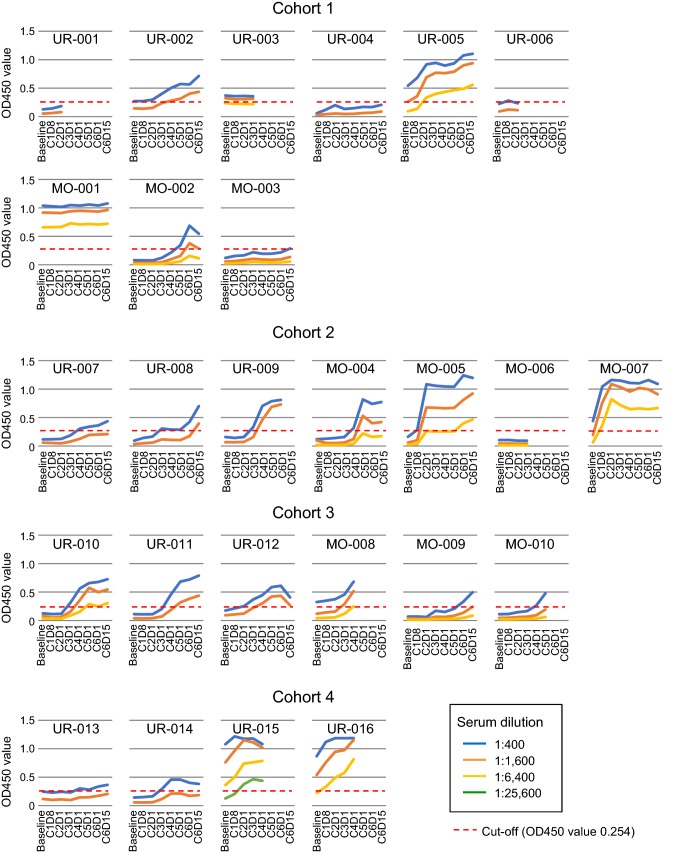
NY-ESO-1-specific IgG response. Anti-NY-ESO-1 IgG was assayed by ELISA at serum dilutions of 1:400 (blue line), 1:1,600 (orange line), 1:6,400 (yellow line) and 1:25,600 (green line). Serum was collected at baseline, course 1 day 8 (C1D8), course 2 day 1 (C2D1), course 3 day 1 (C3D1), course 4 day 1 (C4D1), course 5 day 1 (C5D1), course 6 day 1 (C6D1) and course 6 day 15 (C6D15). The cutoff for OD 450 absorption was 0.254 (red dotted line)

An antibody response was observed in 5 of 9 patients (56%) in cohort 1 (CHP-NY-ESO-1 at 100 µg) and 16 of 17 patients (94%) in cohorts 2–4 (CHP-NY-ESO-1 at 200 µg) (*p* = 0.040) (Table 3, Fig. 1). Among the patients who received more than 3 vaccinations, an antibody response was observed in 4 of 7 patients (57%) in cohort 1 and in 16 of 16 patients (100%) in cohorts 2–4.

### Cytokine analysis

**Fig. 2:**
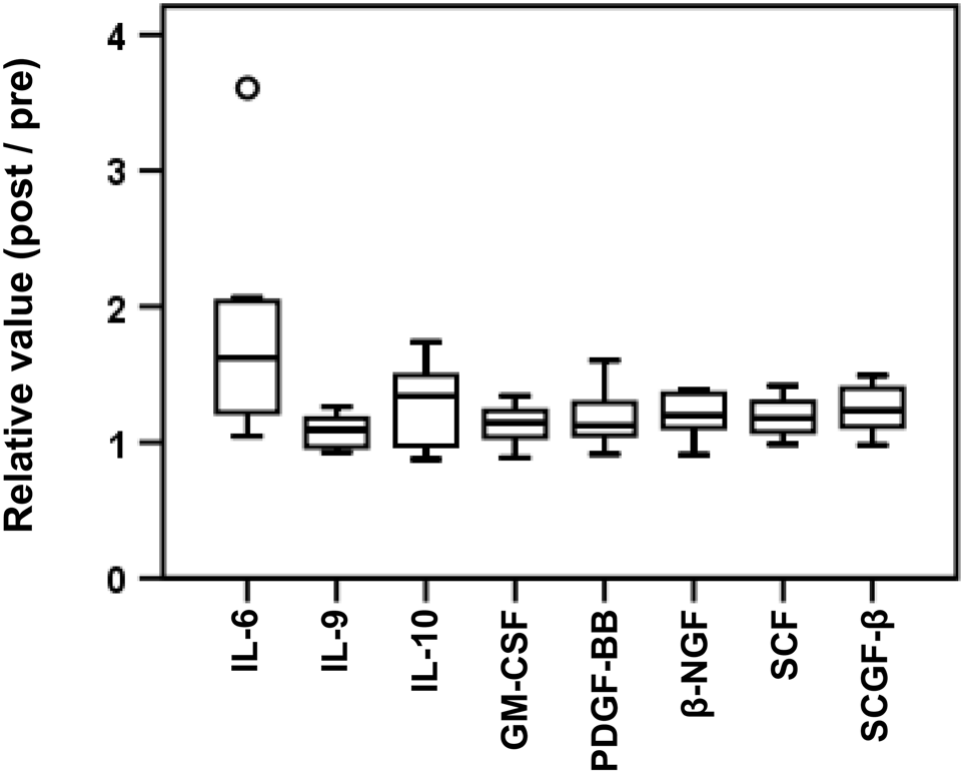
Cytokine analyses. Serum cytokine changes from baseline to 6 h after the first vaccination was assessed. To exclude an effect of the NY-ESO-1 dose, we assessed samples from patients enrolled in cohorts 2–4 (CHP-NY-ESO-1 200 µg with MIS416 200–600 µg). Only those cytokines for which levels significantly changed are shown. The following cytokines had levels that significantly changed from baseline to 6 h after the 1st vaccination: IL-6 (*p* = 0.033), IL-9 (*p* = 0.048), IL-10 (*p* = 0.018), GM-CSF (*p* = 0.020), PDGF-BB (*p* = 0.049), β-NGF (*p* = 0.005), SCF (*p* = 0.003) and SCGF-β (*p* = 0.003)

We hypothesized that the MIS416 adjuvant affected serum cytokine levels in the early phase. The changes in serum cytokine/chemokine levels that occurred from baseline to 6 h after the 1st vaccination were determined using a multiplex assay. Sixteen patients’ samples were assessable. To exclude an effect of NY-ESO-1 dose, we assessed samples from patients enrolled in cohorts 2–4. Compared with baseline values, the levels of IL-6, IL-9, IL-10, GM-CSF, PDGF-BB, β-NGF, SCF and SCGF-β were significantly higher at 6 h after the first vaccination (Fig. 2). In contrast, no cytokines significantly decreased. With regard for the MIS dose, a comparison of cohorts 2, 3 and 4 revealed that the level of IL-17 tended to increase as the MIS416 dose increased (Supplementary Figure 1).

### T cell response

Patients whose PBMCs were available for assessing T cell responses are listed in Table 3. The T cell response was assessed in two patients per cohort, for a total of 8 patients. No increase in NY-ESO-1-specific IFN-γ secreting CD8^+^ T cells was detected after vaccination. In contrast, the number of NY-ESO-1-specific IFN-γ-secreting CD4^+^ T cells increased in 2 patients after vaccination. One of these 2 patients, Pt ID UR-008, did not show progression for 5 years, as mentioned above, but did exhibit a NY-ESO-1-specific CD4^+^ T cell response (Table 3 and Supplementary Fig. 2a).

### Quality of life

In total, 11 patients were assessed for QOL. There was no significant difference in any score, including the 5 functional scales, 3 symptom scales, and global health and QOL scale, during treatment (Supplementary Fig. 3).

### Preclinical study of CHP-NY-ESO-1 + MIS416 with a PD-1 inhibitor

**Fig. 3:**
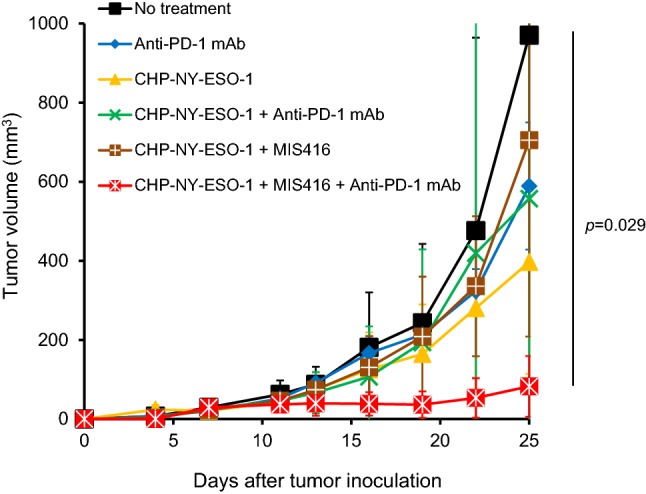
Addition of the PD-1 inhibitor to CHP-NY-ESO-1 + MIS416 in the NY-ESO-1-transfected tumor–bearing mouse model. BALB/c mice subcutaneously inoculated with 1 × 10^6^ human NY-ESO-1-transfected murine colon tumor cell line CT26 cells were treated with CHP-NY-ESO-1 alone, anti-PD-1 mAb (clone RMP1-14) alone, CHP-NY-ESO-1 + MIS416, CHP-NY-ESO-1 + anti-PD-1 mAb, or CHP-NY-ESO-1 + MIS416 + anti-PD-1 mAb (n = 4 per group). CHP-NY-ESO-1 (40 μg/mouse) and MIS416 (250 μg/mouse) were administered subcutaneously on days 1 and 7. The anti-PD-1 monoclonal antibody (clone RMP1-14, 150 μg/mouse) was administered intraperitoneally on days 1, 4, 7, 9, 13, 16 and 19. Compared with the no-treatment group, only the CHP-NY-ESO-1 + MIS416 + anti-PD-1 mAb group showed significant tumor growth suppression (*p*  = 0.029)

To determine whether CHP-NY-ESO-1 exerts anti-tumor activity in an animal model, we assessed vaccine efficacy by adding an anti-PD-1 monoclonal antibody (mAb) to the CHP-NY-ESO-1 + MIS416 group. Treatment with CHP-NY-ESO-1, anti-PD-1 mAb, CHP-NY-ESO-1 + anti-PD-1 mAb or CHP-NY-ESO-1 + MIS416 could not suppress tumor growth, whereas treatment with CHP-NY-ESO-1 + MIS416 + anti-PD-1 mAb induced significant tumor growth suppression (Fig. 3).

## Discussion

CHP-NY-ESO-1 is a safe and promising cancer vaccine [21-25]. We expected the addition of MIS416 to make CHP-NY-ESO-1 more efficient without compromising safety. In this study, CHP-NY-ESO-1 with adjuvant MIS416 200–400 μg showed acceptable safety and tolerability. Grade 3 hypertension was observed in five patients (19%). In multiple sclerosis (MS) patients who were administered MIS416 intravenously, vascular disorders, including hypertension, were observed in 52.5% of the patients [34]. In the higher MIS416 dose group (500–600 μg), the frequency of vascular disorders was 83.3%; these included diastolic hypertension in 50.0%, hypertension in 50.0%, and systolic hypertension in 66.7% of the patients. These results indicate that MIS416 may cause vascular AEs. The exact mechanism has not yet been clarified. In our study, among the 5 patients with grade 3 hypertension, 4 had prostate cancer. A previous meta-analysis showed that there was a relationship between hypertension and the risk of prostate cancer [35]. Because some cytokines can elevate blood pressure [36], prostate cancer patients with hypertension at baseline might be more sensitive to MIS416-induced cytokines. In line with the results of a previous study [25], we found that the antibody response was stronger in patients who received CHP-NY-ESO-1 200 µg than in those who received CHP-NY-ESO-1 100 µg (Table 3). The antibody response rate and cycle number in the seropositive group were similar between the report by Kageyama et al. (53.8% and a median of 2 cycles in the CHP-NY-ESO-1 100 μg group; and 100% and a median of 2 cycles in the CHP-NY-ESO-1 200 μg group, respectively) and our study (56% and a median of 3 cycles in the CHP-NY-ESO-1 100 μg + MIS416 200 μg group; and 94% and a median of 2 cycles in the CHP-NY-ESO-1 200 μg + MIS416 200–600 μg group). The IgG subtype was also considered in our study. Karbach et al. reported that when CpG was added to the NY-ESO-1 vaccine, IgG1 and IgG3 responses were induced [5]. To clarify the effect of MIS416, we compared the titers of IgG1, IgG2 and IgG3 in serum obtained from patients in cohorts 2–4 (CHP-NY-ESO-1 200 µg with MIS416) to those reported in 8 patients enrolled in a previous study by Kageyama et al. of CHP-NY-ESO-1 200 µg without an adjuvant [25]. One patient in cohort 3 of our study was vaccinated twice and had negative seroconversion, and this patient was excluded from the IgG analysis. CHP-NY-ESO-1 induced a prominent IgG1 response with increased IgG2 and IgG3 titers. However, adding MIS416 seemed to suppress IgG1, 2 and 3 responses (Supplementary Fig. 4a). While some patients did use steroids, NY-ESO-1-specific IgG1 titers were not affected by steroid use in cohorts 2–4 (Supplementary Fig. 4c). The patients’ QOL was well-maintained during vaccination with CHP-NY-ESO-1 with MIS416 (200 and 400 µg) (Supplementary Fig. 3). However, CHP-NY-ESO-1 vaccination with 600 μg MIS416 was not well-tolerated, and tumor shrinkage was not observed in this group. Furthermore, the immune response stimulated by CHP-NY-ESO-1 was not enhanced by increasing the dose of MIS416.

MIS416 skewed the Th1 response in a mouse model [10]. In vitro, MIS416 was readily internalized by human myeloid and plasmacytoid DCs, resulting in cytokine secretion and cell activation/maturation. In this study, the subcutaneous injection of MIS416 caused IL-6 and IL-10 levels to be increased at 6 h after the first vaccination (Fig. 2). CHP-NY-ESO-1 vaccination showed CD4^+^ and CD8^+^ T cell responses in previous studies [22-24]. Unfortunately, in this study, we did not find that CD4^+^ Th1 cell and CD8^+^ T cell responses were enhanced. As mentioned above, we compared serum IgG1, IgG2 and IgG3 titers between samples obtained from patients in cohorts 2–4 (CHP-NY-ESO-1 200 μg with MIS416) and those obtained from 8 patients enrolled in a previous study by Kageyama et al. in which the patients received CHP-NY-ESO-1 200 μg without an adjuvant [25]. The anti-NY-ESO-1 IgG1 response was also attenuated as the dose of MIS416 increased (Supplementary Fig. 4b). The NY-ESO-1-specific IgG1 titer was significantly lower in patients who received MIS416 600 μg than in those who received the vaccine without MIS416. Two main causes might lead to these unexpected results. One cause is species-specific differences [37]. Although MIS416 suppressed IL-17 in an MS mouse model [38], this result has not been confirmed in humans with MS [34]. In our study, IL-17 levels tended to increase as the dose of MIS416 increased (Supplementary Fig. 1). When the effects of different CHP-NY-ESO-1 doses were compared, no significant change in the serum cytokine levels was observed with the exception of IL-13 (Supplementary Fig. 5). Based on the findings in a mouse model, we could not predict whether the human innate immune system would be activated by the TLR9 and NOD2 signals included in MIS416, leading to an adaptive immune response. Another cause is the paradoxical effect of MIS416. IL-10 generally acts as an immunosuppressive cytokine but can enhance the CD8^+^ T cell response at higher doses [39, 40]. Although IL-10 levels were increased in this study (Fig. 2), the change in absolute concentration was small (median + 1.3 pg/mL, Supplementary Table 4). In vitro, MIS416 stimulated PBMCs, causing them to secrete IL-6, IL-10, IFN-γ, TNF-α and IL-1β and exhibit an inverse dose–response to MIS416 at 5, 20 and 50 µg/mL [41]. In an MS mouse model, a high dose of MIS416 (100 µg/mouse) resulted in the systemic suppression of pro-inflammatory cytokine levels [38]. In addition, in humans, in a clinical trial in which a TLR9 stimulant was applied, the higher dose group had a lower response rate than was observed in the lower dose group (≤ 2 mg 80%, 8 mg 38%) [42]. These findings suggest that TLR stimulation may suppress the innate response in a dose-dependent manner. However, it is not possible to directly compare data obtained using MIS416 preclinical and human studies since the route of administration was different between the present and previous studies, and this is an important factor. In a previous study of MS patients, MIS416 was administered intravenously with the expectation that it would move to the liver and act as an immunosuppressive agent; in contrast, in this study, MIS416 was administered subcutaneously with the expectation that it would exert an immune stimulatory effect in a draining lymph node [10].

There are several limitations of this study. First, there are a variety of cancer types, and we cannot exclude differences in prior systemic therapy and immunogenicity in different cancers. Second, the effect of steroids cannot be ruled out as steroid use is an important issue in cancer immunotherapy. For example, in patients with prostate cancer, steroids may elicit anti-prostate cancer effects. Combined systemic therapy with steroids is commonly administered to patients with prostate cancer refractory to castration [43-46]. In this study, steroid use (≤ 20 mg equivalent of prednisolone/day) was allowed. Eight patients (31%) used DEX, with 1 mg/day used by 3 patients and ≤ 0.5 mg/day used by 5 patients. All 8 patients were prostate cancer patients who received DEX at enrollment and throughout this study. Among these 8 patients, 38% achieved SD. In the cytokine analysis, steroid use was found to suppress changes in serum G-CSF and CCL2 levels (Supplementary Fig. 6). However, the measured values showed no significant differences. Steroid use also did not affect NY-ESO-1-specific IgG1 titers (Supplementary Fig. 4c). Interestingly, one of the 8 patients who were enrolled in cohort 2 experienced no progression for 5 years and showed a NY-ESO-1-specific CD4^+^ T cell response. Cancer vaccines are known to be effective in prostate cancer, as shown in studies using Sipuleucel-T [47, 48]. Yoshimura et al. [49] reported that patients who received a peptide vaccine plus DEX had longer PSA progression-free survival than was observed in patients who received DEX alone. Although we cannot deny that immune suppression was induced by steroids, these findings suggest that the induction of the NY-ESO-1-specific T cell response shows promising effects for prostate cancer patients, even among patients undergoing low-dose systemic steroid treatment.

It has been assumed that the appropriate clinical use of an immune-stimulating adjuvant enhances the anti-tumor effects of cancer vaccines. Based on this idea, a number of cancer vaccine clinical trials have been conducted worldwide; however, almost all of them have produced disappointing results. Melanoma-associated antigen (MAGE)-A3, a cancer-testis antigen, is one of the most promising targets for cancer vaccines [50]. Recombinant MAGE-A3 vaccination with the AS15 immunostimulant, which contains CpG, a TLR9 stimulant, was assessed in patients with melanoma and non-small cell lung cancer but did not produce a survival benefit [51, 52]. This finding suggests that cancer vaccines cannot be developed as monotherapies. In our study, no complete or partial response was observed. The combination of CHP-NY-ESO-1 + MIS416 + anti-PD-1 mAb exerted a significant tumor growth suppression effect in the mouse model (Fig. 3). The addition of anti-PD-1 mAb activates not only NY-ESO-1-specific T cells but also other tumor antigen-reactive T cells. A combination therapy that includes a cancer vaccine with the proper adjuvant and an immune checkpoint inhibitor may confer clinical anti-tumor effects.

In conclusion, CHP-NY-ESO-1 with MIS416 200–400 µg was safe and tolerable but did not induce adequate immune or clinical responses. As a next step, we plan to conduct a clinical trial of a combination therapy that includes a cancer vaccine, an adjuvant and an immune checkpoint inhibitor.

## Electronic supplementary material

Below is the link to the electronic supplementary material.

Supplementary file1 (PDF 1012 kb)

## Abbreviations

ALT: Alanine aminotransferase
bFGF: Basic fibroblast growth factor
CCL: C–C motif chemokine ligand
CHP: Cholesteryl pullulan
CRPC: Castration-resistant prostate cancer
CTACK: Cutaneous T-cell-attracting chemokine
CXCL: C–X–C motif chemokine ligand
DEX: Dexamethasone
DLT: Dose-limiting toxicity
ECOG: Eastern Cooperative Oncology Group
EORTC: European organization for research and treatment of cancer
GAPDH: Glyceraldehyde-3-phosphate dehydrogenase
GRO: Growth-related oncogene
HGF: Hepatocyte growth factor
LIF: Leukemia inhibitory factor
MAGE: Melanoma-associated antigen
M-CSF: Macrophage colony-stimulating factor
MIF: Macrophage migration inhibitory factor
MS: Multiple sclerosis
MTD: Maximum tolerated dose
NGF: Nerve growth factor
NOD: Nucleotide-binding oligomerization domain
NY-ESO-1: New York esophageal squamous cell carcinoma 1
PD: Progressive disease
PDGF: Platelet-derived growth factor
PS: Performance status
QOL: Quality of life
qRT-PCR: Quantitative real-time PCR
RECIST: Response evaluation criteria in solid tumors
SAE: Severe adverse event
s.c.: Subcutaneously
SCF: Stem cell factor
SCGF: Stem cell growth factor
SD: Stable disease
SDF: Stromal cell-derived factor
SEREX: Serological expression cloning
UC: Urothelial cancer
