# Supplementary material for: First-in-human phase I clinical trial of the NY-ESO-1 protein cancer vaccine with NOD2 and TLR9 stimulants in patients with NY-ESO-1-expressing refractory solid tumors
Source: Cancer Immunol Immunother. 2020 Jan 24;69(4):663–75. doi: 10.1007/s00262-020-02483-1 (PMC7113205; doi:10.1007/s00262-020-02483-1)
Supplement: Supplementary file 1 — Supplementary file1 (PDF 1012 kb) [file 262_2020_2483_MOESM1_ESM.pdf]

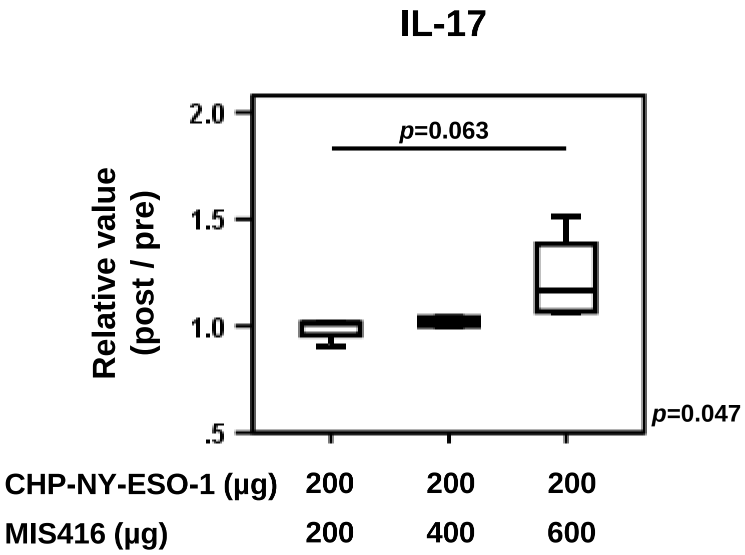

Supplementary Fig. 1. Cytokine analyses: Comparison with MIS416 doses. IL-17 tended to increase in a MIS416 dose-dependent manner. The *P* value for the comparison between patients receiving MIS416 200 μg and those receiving MIS 600 μg was 0.021, but this comparison was not significant after Bonferroni correction (*p*=0.063). The CHP-NY-ESO-1 dose was 200 μg.

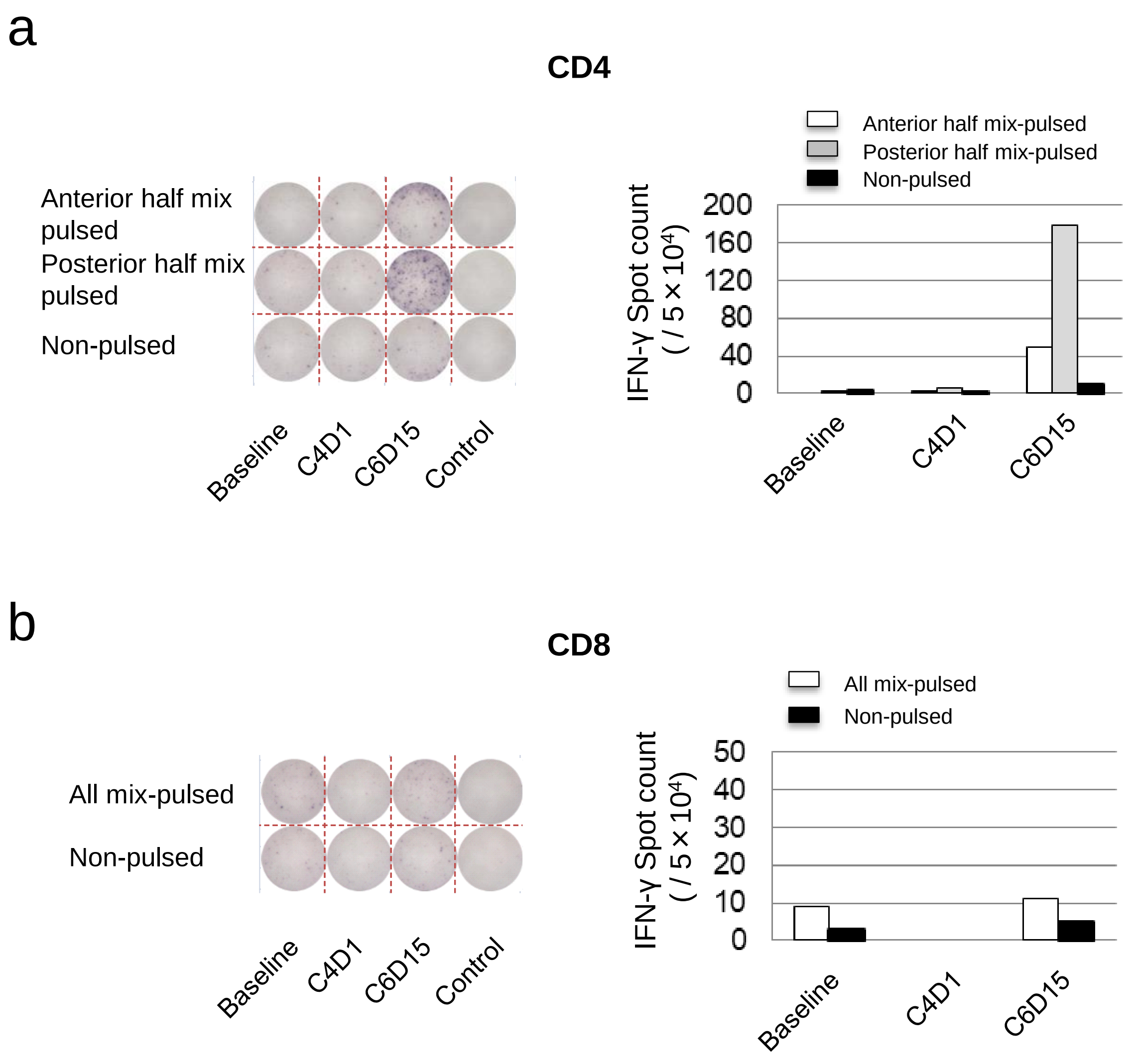

Supplementary Fig. 2. T cell response in patient ID UR-008. IFN-γ-secreting T cells at baseline and on treatment phase course 4 day 1 and treatment phase course 6 day 15 were assessed by ELISPOT assay. (a) In total, 5×10^4 CD4+ T cells were cultured with 1×10^5 irradiated CD4- CD8- peripheral blood mononuclear cells pulsed with an anterior half mix, a posterior half mix or no peptide. (b) In total, 5×10^4 CD8+ T cells were cultured with 1×10^5 irradiated CD4- CD8- peripheral blood mononuclear cells, and the whole mix was then pulsed (anterior half mix plus posterior half mix) or no peptide.

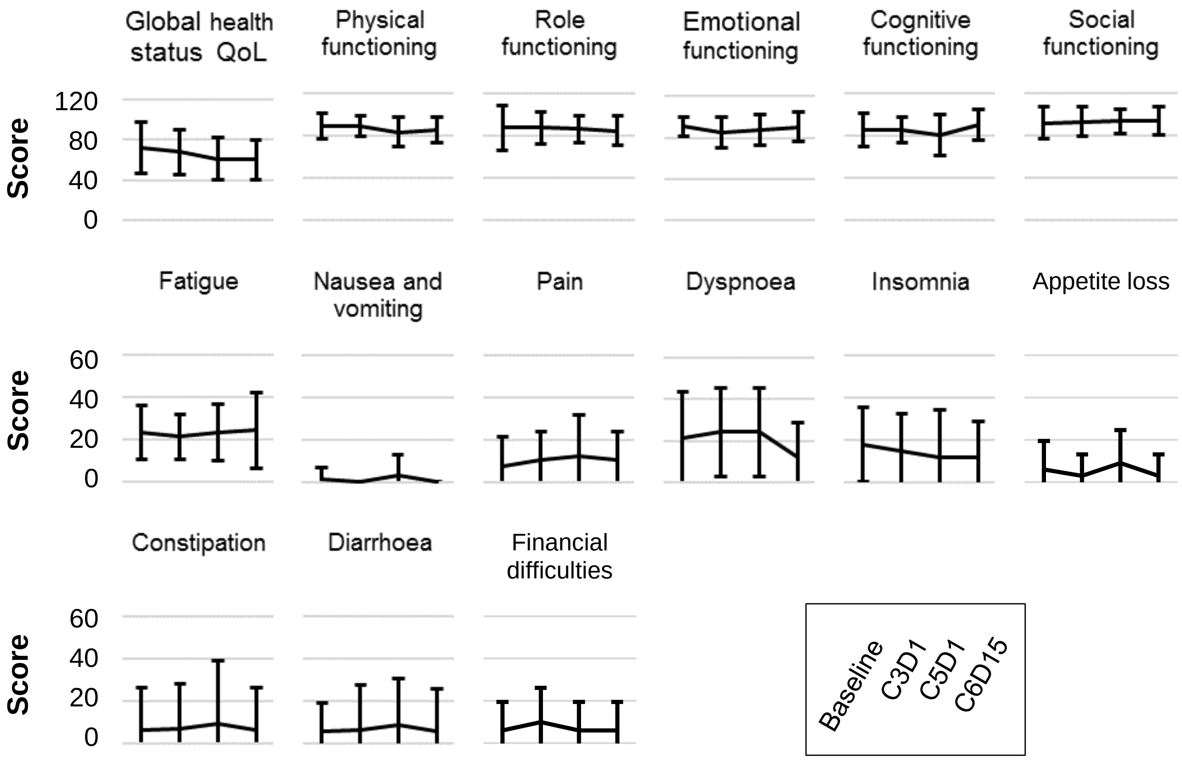

Supplementary Fig. 3. QOL assessment using the EORTC QLQ-C30. The EORTC QLQ-C30 was assessed at baseline and on treatment phase course 3 day 1, treatment phase course 5 day 1, and treatment phase course 6 day 15. There were no statistically significant differences in any scores during treatment.

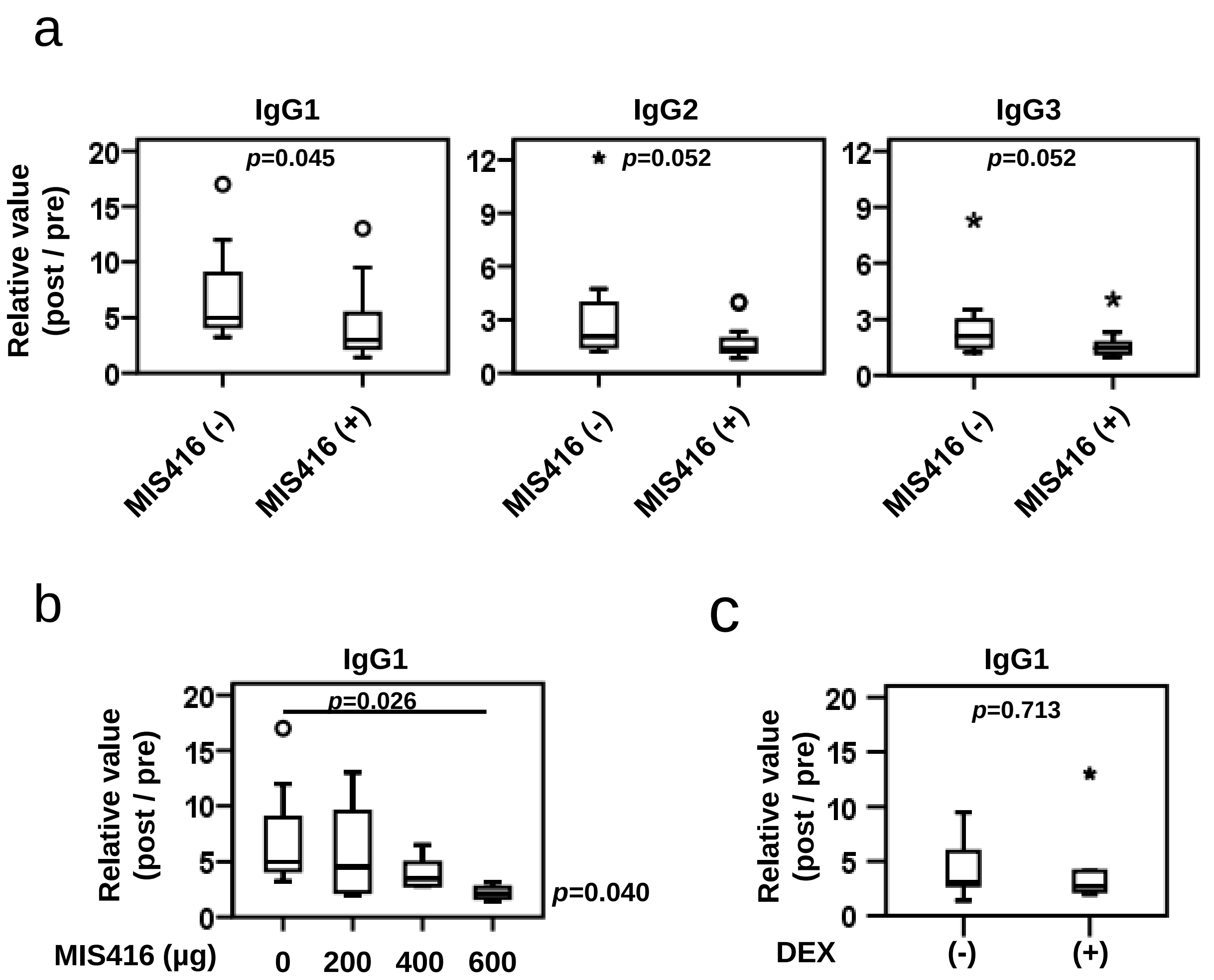

Supplementary Fig. 4. NY-ESO-1-specific IgG analyses. Patients administered CHP-NY-ESO-1 200 µg were included in these analyses. (a) IgG1, 2 and 3 titers in serum obtained from patients administered CHP-NY-ESO-1 200 µg with MIS416 were compared to those in serum samples obtained from patients administered CHP-NY-ESO-1 200 µg without MIS416. The addition of MIS416 to CHP-NY-ESO-1 resulted in a significant decrease in NY-ESO-1-specific IgG1 titers. (b) NY-ESO-1-specific IgG1 titers were compared between patients administered CHP-NY-ESO-1 200 µg with MIS 0 µg, MIS416 200 µg, MIS416 400 µg, or MIS416 600 µg. Kruskal-Wallis ANOVA was used to compare data obtained in the 4 groups. The P value for the comparison between patients administered MIS 0 µg and those administered 600 µg was 0.026 after Bonferroni correction. (c) The NY-ESO-1-specific IgG 1 titers of patients in cohorts 2-4 were assessed with or without steroid use. Fold changes were calculated as post-/pre-OD values. The OD value at the dilution first judged ‘positive’ or ‘enhanced’ was used as the ‘post-OD’ value, and the OD value of the same dilution at baseline was used as the ‘pre-OD’ value. Circles and asterisks indicate outliers.

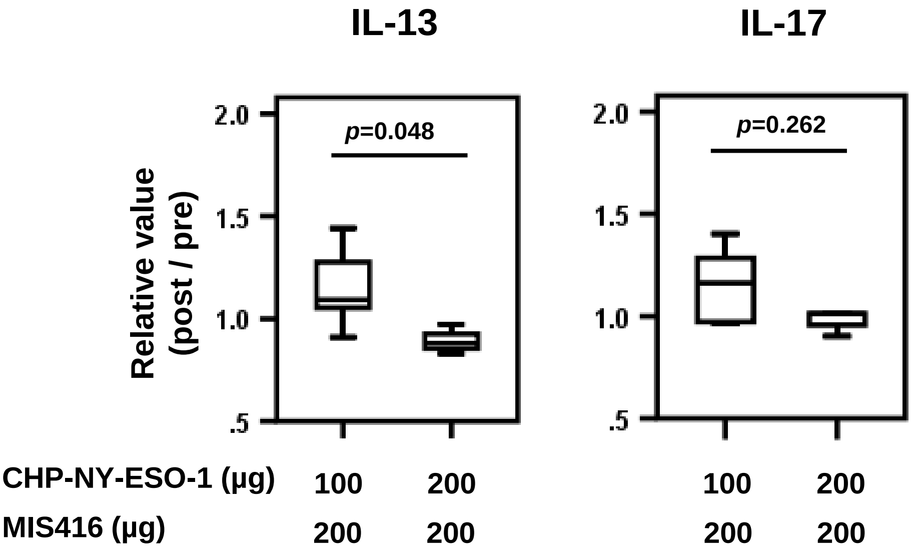

Supplementary Fig. 5. Cytokine analyses: Comparison with CHP-NY-ESO-1 doses. Except for IL-13, there were no differences in serum cytokine values between cohort 1 (CHP-NY-ESO-1 100 µg + MIS416 200 µg) and cohort 2 (CHP-NY-ESO-1 200 µg + MIS416 200 µg).

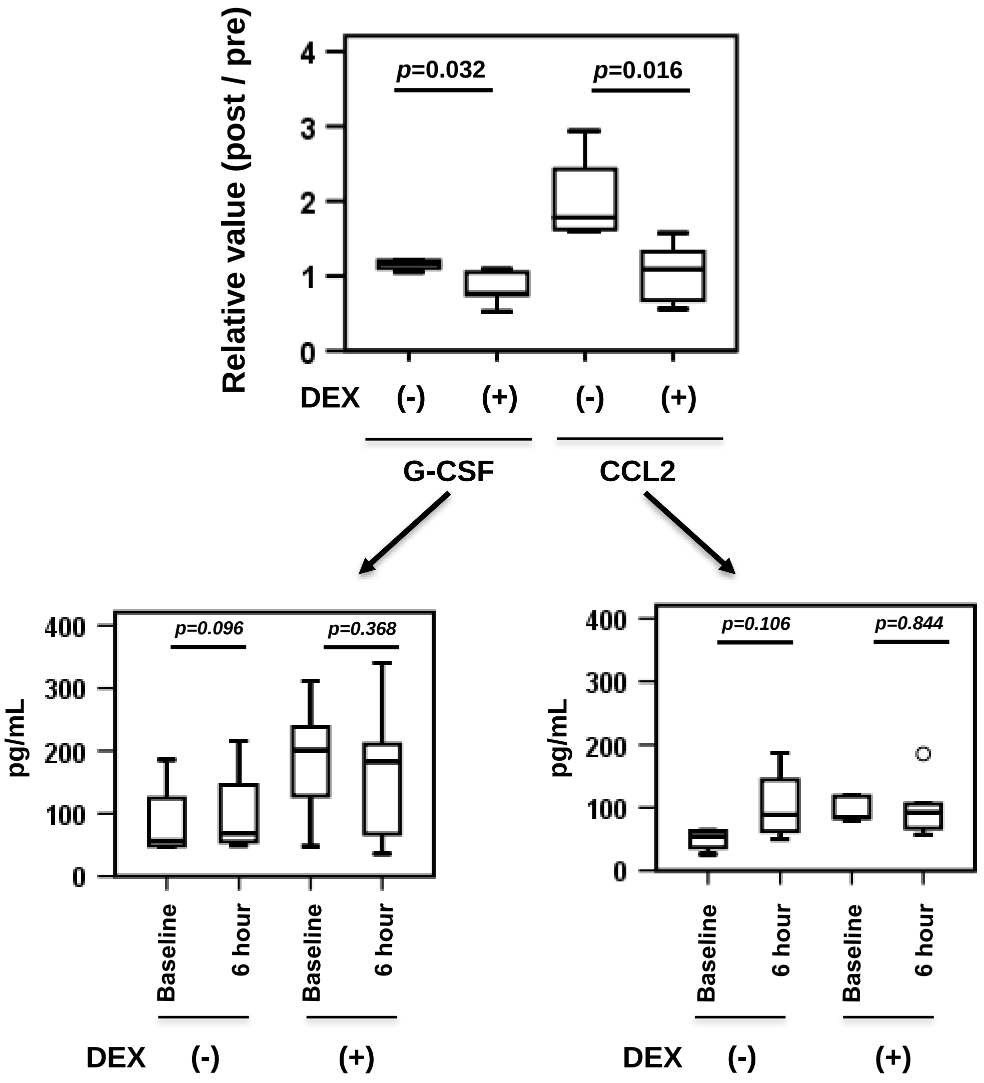

Supplementary Fig. 6. Cytokine analyses: Comparison of DEX use and nonuse. Serum cytokine changes were assessed from baseline to 6 hours after the 1st vaccination. To exclude an effect of the NY-ESO-1 dose, we assessed samples obtained from patients enrolled in cohorts 2-4 (CHP-NY-ESO-1 200 µg with MIS416 200-600 µg). Only the relative values of G-CSF and CCL2 were significantly lower in DEX users than in nonusers. However, the measured values showed no significant differences. A paired t-test was used to analyze the measured values. Circles and asterisks indicate outliers.

Supplementary Table 1. The details of patient characteristics.

Cohort	Pt ID	Age	Sex	Tumor type	Prior anticancer therapy				NY-ESO-1 expression (IHC, %)
					Chemo therapy	Hormonal therapy	Radio therapy	Surgery	
1	UR-001	78	M	Prostate	Yes	Yes	No	No	10
	UR-002	82	M	Prostate	Yes	Yes	No	No	100
	UR-003	62	M	Bladder	Yes	No	No	Yes	5
	UR-004	75	M	Prostate	Yes	Yes	No	No	60
	UR-005	72	F	Renal pelvis	Yes	No	No	Yes	10
	UR-006	84	M	Urinary duct	Yes	No	No	No	5
	MO-001	64	F	Ovary	Yes	No	No	Yes	80
	MO-002	48	M	Synovial sarcoma	Yes	No	Yes	Yes	95
	MO-003	60	F	Urinary duct	Yes	No	No	Yes	5
2	UR-007	74	M	Prostate	Yes	Yes	No	No	100
	UR-008	73	M	Prostate	Yes	Yes	No	No	100
	UR-009	82	M	Prostate	Yes	Yes	Yes	No	100
	MO-004	49	F	Esophagus	Yes	No	Yes	No	60
	MO-005	74	M	Prostate	Yes	Yes	No	Yes	100
	MO-006	67	M	Sarcoma	Yes	No	Yes	Yes	70
	MO-007	53	F	Synovial sarcoma	Yes	No	Yes	Yes	10
3	UR-010	75	M	Prostate	Yes	Yes	No	No	40
	UR-011	48	M	Prostate	Yes	Yes	Yes	No	5
	UR-012	62	M	Prostate	No	Yes	Yes	Yes	10
	MO-008	41	M	Rectum	Yes	No	No	No	100
	MO-009	36	F	Synovial sarcoma	Yes	No	No	Yes	100
	MO-010	53	M	Synovial sarcoma	Yes	No	No	Yes	95
4	UR-013	81	M	Prostate	Yes	Yes	No	Yes	10
	UR-014	67	M	Prostate	Yes	Yes	No	Yes	10
	UR-015	73	M	Bladder	Yes	No	No	Yes	10
	UR-016	77	M	Prostate	Yes	Yes	No	Yes	80

Supplementary Table 2. Prostate cancer patient characteristics.

Cohort	Pt ID	History of orchiectomy	Concurrent cheminical castration	Gleason's score	Steroid Use (/day)	Response after the treatment phase
1	UR-001	No	Yes	8	DEX 1mg	N/A
	UR-002	No	No	7	None	SD §
	UR-004	No	No	7	DEX 0.5mg	SD
2	UR-007	No	Yes	9	DEX 1mg	PD
	UR-008	No	Yes	8	DEX ≤0.5mg	SD §
	UR-009	No	No	9	None	PD
	MO-005	Yes	No	9	DEX 0.5mg	SD §
3	UR-010	No	Yes	8	DEX 1mg	PD
	UR-011	No	Yes	10	None	SD §
	UR-012	Yes	No	8	None	PD
4	UR-013	Yes	No	8	DEX 0.5mg	PD
	UR-014	Yes	No	9	DEX 0.5mg	PD
	UR-016	Yes	No	9	None	PD

§ Patients who had no measurable lesion at baseline.

Supplementary Table 3. Treatment-related hypertension.

Cohort	Pt ID	Hypertension grade	History of Hypertension	Hypertension grade at baseline
	UR-001			
	UR-002	1	No	
	UR-003			
	UR-004	3	Yes	2
1	UR-005			
	UR-006			
	MO-001			
	MO-002	1	No	
	MO-003			
	UR-007			
	UR-008	3	Yes	2
	UR-009			
2	MO-004			
	MO-005	3	Yes	2
	MO-006			
	MO-007			
	UR-010			
	UR-011			
3	UR-012	3	Yes	2
	MO-008			
	MO-009			
	MO-010			
	UR-013			
4	UR-014	3	Yes	2
	UR-015			
	UR-016	2	Yes	1

Supplementary Table 4. Serum cytokine concentration at 6 hours after the 1st vaccination.

		UR-001	UR-002	UR-003	UR-04	UR-05	UR-006	UR-007	UR-008	UR-009	UR-010	UR-011	UR-012	UR-013	UR-014	UR-015	UR-016
IL-1β	Baseline	3.1	4.06	4.55	4.49	5	2.5	3.33	3.45	3.12	3.62	1.42	1.02	3.44	1.47	1.39	1.23
	6 hours	3.3	5.44	5.62	5.14	4.42	2.37	3.2	12.83	3.18	3.18	1.5	N/A	1.76	1.05	1.42	1.44
IL-1Rα	Baseline	55.2	128.03	137.56	228.02	161.51	86.54	139.24	140.03	111.71	130.58	66.78	22.88	141.76	47.77	61	52.22
	6 hours	63.28	218.15	174.23	345.93	154.81	86.54	138.45	247.2	109.35	121.14	76.78	N/A	69.65	38.71	66.78	61
IL-2	Baseline	8.58	5.09	30.9	19.27	18.48	7.34	16.69	16.69	10.44	10.51	12	5.45	11.29	10.94	13.93	10.04
	6 hours	10.41	13.82	42.42	36.49	16.13	8.76	17.17	17.52	9.49	14.69	13.41	N/A	12.71	10.04	12.18	12.35
IL-4	Baseline	6.2	6.58	8.46	9.07	8.37	20.83	28.24	28.32	26.93	27.26	3.1	2.12	3.02	3.14	3.46	2.9
	6 hours	6.4	9.25	9.47	10.29	7.28	19.88	28.48	26.6	25.12	25.28	3.58	N/A	3.3	2.69	3.66	3.3
IL-6	Baseline	8.41	9.96	11.34	33.5	14.11	15.29	9.25	14.23	11.55	10.54	6.66	4.94	7.52	7.01	10.95	9.75
	6 hours	14.36	31.99	15.55	54.93	17.46	27.54	9.66	13.7	16.6	11.08	24.03	N/A	10.43	14.1	19.79	20.13
IL-7	Baseline	12.01	10.89	14.52	16.62	15.75	12.71	19.7	19.45	22.43	20.45	10.83	4.99	5.85	7.06	8.49	9.31
	6 hours	9.65	15.4	15.85	21.96	11.87	10.21	26.7	16.56	23.53	16.56	10.13	N/A	7.06	5.61	7.06	11.75
IL-8	Baseline	39.75	36.21	41.37	33.01	41.57	39.55	35.4	40.98	36.16	37.03	15.88	14.43	20.1	16.35	12.96	17.54
	6 hours	27.74	42.56	46.7	43.85	39.55	54.09	29.66	125.54	30.95	32.14	14.91	N/A	16.59	12.71	16.35	17.77
IL-9	Baseline	19.46	29.19	21.27	25.85	48.07	263.22	54.78	58.7	44.6	50.27	20.53	11.79	17.98	19.34	20.87	13.09
	6 hours	19.69	43.41	26.03	38.23	53.18	287.32	50.86	68.94	41.49	50.27	25.93	N/A	21.12	20.7	23.23	15.41
IL-10	Baseline	5.31	9.63	11.87	11.82	8.4	5.32	8.61	9.06	5.14	8.2	2.53	1.43	5.83	0.88	2.43	1.7
	6 hours	4.23	14.18	15.77	16.11	6.66	6.72	8.93	12.82	6.45	7.48	4.4	N/A	5.1	1.27	3.75	2.43
IL-12(p70)	Baseline	15.16	24.15	59.87	25.64	33.26	15.26	29.08	23.05	18.64	23.27	33.68	14.28	66.77	25.51	49.2	37.59
	6 hours	13.85	32.41	69.61	31.29	29.4	19.39	25.45	28.52	16.94	21	43.75	N/A	52.96	20.9	50.32	37.59
IL-13	Baseline	7.55	11.98	15.04	15.04	11.29	8.8	16.58	18.83	14.36	12.63	5.3	3.24	8.55	5.95	5.74	5.52
	6 hours	7.95	15.3	21.68	15.96	10.27	9.91	16.12	16.58	11.92	13.53	4.86	N/A	7.01	9.05	5.95	7.43
IL-15	Baseline	OOR <	OOR <	OOR <	14.23	OOR <	OOR <	9.51	12.41	OOR <	4.65	4.75	3.03	4.57	5	3.78	2.45
	6 hours	OOR <	OOR <	OOR <	37.95	OOR <	OOR <	10.2	12.24	OOR <	16.43	6.96	N/A	6.48	5.67	4.66	5.34
IL-17	Baseline	82.86	83.46	109.81	116.23	97.89	256.02	416.8	449.78	309.27	418.49	33.63	16.53	21.81	31.34	27.07	24.44
	6 hours	80.59	117.04	140.88	145.79	104.52	246.9	421.87	457.41	279.27	435.39	33.63	N/A	32.97	33.3	29.04	30.68
Eotaxin	Baseline	168.46	140.6	189.73	368.81	145.77	500.47	1301.95	817.65	659.12	715.21	213.01	157.77	387.36	190.11	155.67	144.96
	6 hours	142.12	154.4	211.8	500.09	147.37	572.82	698.19	943	674.4	650.64	240.43	N/A	303.92	209.26	152.49	176.25
bFGF	Baseline	41.53	46.18	45.09	37.97	53.09	65.69	87.05	106.2	81.2	109.09	60.1	44.75	55.77	57.03	64.77	58.89
	6 hours	42.88	48.16	55.4	44.72	43.4	60.04	89.8	103.55	73.54	102.49	61.88		57.66	62.46	60.7	61.29
G-CSF	Baseline	24.17	26.84	46.62	38.46	34.5	165.91	200.93	311.46	186.06	238.16	63.27	38.43	127.94	47.97	46.82	50.25
	6 hours	27.48	45.2	56.47	63.13	27.49	182.73	210.75	340.26	215.62	182.73	75.42	N/A	67.4	35.92	49.12	61.17
GM-CSF	Baseline	15.34	35.09	31.71	25.34	44.23	29.01	45.93	54.43	30.89	36.83	36.67	37.56	48.11	44.32	40.21	44.32
	6 hours	21.86	52.31	43.17	38.51	45.24	41.31	53.43	79.9	27.42	35.96	44.9	N/A	59.69	49.86	44.32	59.4
IFN-γ	Baseline	139.55	187.88	197.57	240.11	241.66	131.53	194.78	244.69	166.89	197.64	127.45	70.41	127.45	127.45	94.34	105.69
	6 hours	159.69	242.47	214.49	295	194.59	135.5	195.74	225.06	168.83	192.87	163.33	N/A	163.33	94.34	116.71	116.71
CXCL10	Baseline	719.4	2432.67	1621.22	3375.33	1846.49	27116.73	31466.62	55476.24	33729.61	9844.52	775.7	794.69	1909.62	2310.39	2063.77	1541.66
	6 hours	676.73	2586.01	1710.92	3698.78	1769.71	26092.44	22180.46	67390.59	33634.68	10343.5	853.67	N/A	1305.66	2262.71	2001.98	1679.72
CCL2	Baseline	51.73	14.04	31.37	118.93	33.25	73.85	119.53	117.92	61.74	84.71	47.09	61.82	83.39	79.77	25.86	63.67
	6 hours	46.18	25.51	76.1	152.23	48.72	162.49	67.1	185.62	101.65	92.21	75.19	N/A	56.62	105.6	49.5	187.09
CCL3	Baseline	4.78	6.52	10.08	6.3	7.04	4.66	5.7	6.22	5.36	5.04	4.01	3.7	4.54	3.67	3.39	3.7
	6 hours	4.54	7.15	10.71	6.36	6.65	4.77	5.3	13.71	5.19	*4.89	4.34	N/A	5	3.7	3.48	4.07
PDGF-BB	Baseline	9267.72	6222.81	9110.85	10162.27	3262.63	41500.18	82831.71	67435.03	83717.42	44631.49	4763.03	1336.34	4668.77	5673.68	6105.23	2841.66
	6 hours	9966.71	9703.47	11313.43	15594.24	3954.27	47246.72	75620.98	*108853.23	89399.92	55791.59	6354.56	N/A	5026.27	5867	7147.19	4558.55
CCL4	Baseline	89.28	133.07	127.94	188.17	113.35	123.14	171.34	250.63	165.46	102.2	157.18	85.86	161.23	189.82	170.22	109.82
	6 hours	84.29	171.85	170.18	230.21	157.56	138.91	137.81	578.88	209.4	122.5	170.16	N/A	150.95	179.66	191.01	138
CCL5	Baseline	15800.51	11850.52	31653.29	18425.92	12878.47	31786.23	29697.99	37772.63	30605.81	31889.84	40902.81	4051.76	34281.42	43064.64	10373.28	7637.02
	6 hours	22175.53	13617.55	33186.72	24504.76	12941.27	31868.27	36209.72	31725.88	34732.87	37285.47	30738.07	N/A	42947.22	37493.38	14849.1	24006.48
TNF-α	Baseline	26.85	35.95	49.86	65.78	79.71	33.74	38.92	48.34	44.76	44.76	38.77	27.58	42.5	31.31	35.04	32.56
	6 hours	27.5	57.84	53.5	85.79	77.41	34.07	43.14	45.09	44.44	37.3	38.77	OOR <	41.88	40.01	36.29	37.53
VEGF	Baseline	174.92	180.52	244.13	118.24	112.67	190.09	286.24	291.24	123.49	190.09	115.88	71.2	362.15	129.14	128.08	137.9
	6 hours	208.62	254.32	335.05	251.22	136.24	227.89	277.1	576.34	142.44	265.53	191.8	N/A	240.53	117.47	165.76	202.43
β-NGF	Baseline	52.97	13.38	16.06	40.02	27.97	23.01	17.69	23.65	22.38	18.93	5.44	5.25	10.67	7.57	9.89	6.41
	6 hours	55.81	17.26	33.55	46.14	17.26	30.11	18	33.74	26.86	22.69	7.57	N/A	9.7	10.28	11.84	8.73
IL-18	Baseline	49.05	44.11	48.71	97.55	63.8	116.27	55.11	164.91	140.09	91.33	119.33	74.3	95.49	58.45	113.58	45.69
	6 hours	51.81	68.32	163.37	181.5	53.61	151.69	51.83	405.84	193.01	118.14	141.55	N/A	61.24	61.42	118.85	60.68
GROα	Baseline	253.42	436.13	OOR <	71.01	OOR <	302.54	300.97	726.97	133.54	59.58	50.15	68.35	162.31	64.67	60.81	48.99
	6 hours	277.81	577.96	128.87	258.68	OOR <	423.15	179.5	1095.79	142.88	244.97	109.42	N/A	107.54	109.42	126.47	129.23
IFN-α2	Baseline	86.05	39.29	64.88	98.7	71.09	90.48	66.09	71.92	81.95	62.22	43.82	35.54	82.49	58.85	84.5	60.71
	6 hours	86.05	84.37	90.15	129.7	88.1	74.7	63.01	151.38	77.4	72.63	46.26	N/A	69.25	64.26	81.81	72.4
M-CSF	Baseline	61.82	1.69	OOR <	27.21	14.7	22.87	17.96	16.84	26.79	8.92	6.97	12.25	36.35	18.24	20.04	14.35
	6 hours	67.84	19.56	40.53	72.61	18.31	48.03	22.37	105.19	35.4	25.13	19.76	N/A	28.38	27.29	26.2	30.14
TRAIL	Baseline	44.79	82.58	60.03	179.22	141.72	70.95	84.54	141.83	70.38	117.28	123.9	120.69	191.97	128.72	120.69	111.03
	6 hours	46.05	83.4	117.82	138.45	124.38	68.08	58.9	114.63	77.3	85.7	130.32	N/A	151.17	207.15	132.73	130.32
IL-2Rα	Baseline	138.29	112.98	43.7	135.14	81.75	192.48	102.47	134.61	162.38	99.77	62.04	77.86	89.01	73.21	140.85	87.15
	6 hours	124.1	125.33	135.14	191.44	79.23	228.77	99.77	182.04	189.87	115.93	76	N/A	78.79	83.44	133.46	109.4
SCF	Baseline	393.7	83.91	48.54	38.64	351.89	238.92	127.88	99.99	221.02	105.83	122.11	181.79	178.37	168.89	233.89	158.41
	6 hours	365.75	90.45	146.99	47.15	294.97	303.35	139.4	119.76	257.85	149.86	154.98	N/A	175.74	200.76	248.27	210.04
SDF-1α	Baseline	90.71	64.43	OOR <	165.44	112.91	208.2	194.14	170.55	180.01	282.29	370.23	277.29	603.28	206.81	603.28	309.63
	6 hours	120.31	177.43	176.4	187.25	11.11	161.05	151.52	359.64	226.86	217.55	480.03	N/A	603.28	505.72	801.08	555.45
IL-16	Baseline	511.65	190.35	209.52	234.27	300.23	266.95	216.75	220.8	209.28	70.85	250.89	419.52	3284.06	140.69	400.57	615.24
	6 hours	565.96	433.33	660.67	1140.06	304.76	319.62	88.88	3492.22	191.39	116.31	235.99	N/A	637.43	222.71	619.42	821.77
HGF	Baseline	914.12	637.68	557.05	485.01	599.28	800.16	575.85	542.64	457.88	377.66	335.05	335.89	484.73	350.23	548.42	466.73
	6 hours	1019.71	784.07	1078.48	901.38	803.56	726.45	452.4	1582.46	427.78	380.37	553.61	N/A	426.59	330.84	690.21	466.73
MIF	Baseline	220.39	249.61	124.34	346.73	145.73	760.97	491.4	160.46	139.4	86	65.09	443.94	1284.5	138.74	438.01	161.86
	6 hours	429.87	256.47	1069.97	771.19	227.6	2022.61	141.76	2726.66	201.64	338.22	516.64	N/A	293.74	271.78	777.14	333.2
CTACK	Baseline	2450.11	2140.93	1019.36	3871.68	1769.43	4035.79	2127.49	2353.14	2810.08	1815.08	1063.62	1377.45	1868.97	1994.39	2074.81	1304.72
	6 hours	1827.74	2266.91	2704.08	6270.49	2142	4673.19	1776.24	3045.07	3245.82	2084.78	1740.85	N/A	1775.37	2052.26	2369.15	1647.46
CXCL9	Baseline	3194.45	8644.86	1056.22	4638.78	3132.61	11601.44	5404.03	20671.39	14714.32	2776.47	402.65	796.62	2330.64	1869.37	3557.62	1674.01
	6 hours	3102.17	9467.73	2202.83	6202.62	3218.44	13844.71	4488.59	26619.69	19184.9	3127.65	440.21	N/A	1806.74	2020.03	3756.14	1884.22
SCGF-β	Baseline	31914.38	9047.72	6929.63	14076.87	22572.31	14364.84	43358.3	32691.77	34271.56	23603.23	45523.46	37264.87	59823.39	31706.9	38601.37	24107.59
	6 hours	38289.24	16895.59	30154.75	25890.01	15795.49	23404.73	44890.27	52239.39	42083.22	35234.47	56637.25	N/A	58562.09	37857.92	50730.07	35695.27
IL-1α	Baseline	OOR <	1.83	OOR <	0.26	3.45	OOR <	OOR <	OOR <	OOR <	OOR <	0.04	OOR <	0.88	0.3	0.95	0.3
	6 hours	OOR <	2.66	0.08	0.06	2.75	OOR <	OOR <	OOR <	OOR <	OOR <	0.61		0.71	0.71	1.63	0.68
IL-3	Baseline	OOR <	OOR <	OOR <	OOR <	OOR <	OOR <	OOR <	OOR <	OOR <	OOR <	7.59	28.63	69.38	36.05	108.12	36.05
	6 hours	OOR <	OOR <	OOR <	70.66	OOR <	OOR <	OOR <	58.12	OOR <	OOR <	36.05		50.3	57.22	127.97	55.84
IL-5	Baseline	4.25	7.55	13.18	10.57	11.58	4.77	7.92	8.48	6.46	7.13	OOR <	OOR <	OOR <	OOR <	OOR <	OOR <
	6 hours	4.88	11.08	15.14	13.23	9.38	5.65	7.66	6.77	5.97	5.33	OOR <	OOR <	OOR <	OOR <	OOR <	OOR <
CCL7	Baseline	46.56	OOR <	5.8	63.78	77.93	OOR <	OOR <	OOR <	OOR <	OOR <	OOR <	OOR <	OOR <	OOR <	0.2	OOR <
	6 hours	40.68	OOR <	31.69	74.99	42.91	OOR <	OOR <	83.29	OOR <	OOR <	OOR <		23.56	OOR <	38.41	17
IL-12p40	Baseline	OOR <	OOR <	OOR <	OOR <	OOR <	OOR <	OOR <	OOR <	OOR <	OOR <	OOR <	OOR <	OOR <	OOR <	OOR <	OOR <
	6 hours	OOR <	OOR <	OOR <	OOR <	OOR <	OOR <	OOR <	OOR <	OOR <	OOR <	OOR <	OOR <	OOR <	OOR <	196.03	OOR <
TNF-β	Baseline	0.2	OOR <	OOR <	0.2	0.99	0	OOR <	0.21	0.68	1.55	OOR <	OOR <	1.73	0.66	1.73	OOR <
	6 hours	0.33	OOR <	1.92	1.2	1.81	1.68	0	3.17	2.06	2.71	0.35		1.29	0.25	2.05	1.73
LIF	Baseline	OOR <	OOR <	OOR <	OOR <	OOR <	2.87	6.93	10.22	OOR <	6.93	0.57	4.41	12.54	5.5	11.52	4.13
	6 hours	OOR <	OOR <	OOR <	38.44	OOR <	13.14	13.27	43.07	12.21	21.15	6.31		11.52	6.84	17.31	9.72

OOR<, Out of Range below. (pg/ml)
